# Treatment options for unresectable hepatocellular carcinoma with hepatitis virus infection following sorafenib failure

**DOI:** 10.1007/s00262-022-03324-z

**Published:** 2022-11-28

**Authors:** Xiaomi Li, Xiaoyan Ding, Wei Li, Jinglong Chen

**Affiliations:** Department of Cancer Center, Beijing Ditan Hospital, Capital Medical University, Beijing, 100015 China

**Keywords:** Hepatocellular carcinoma, Sorafenib, Tyrosine kinase inhibitor, Immune checkpoint inhibitor, Second-line treatment

## Abstract

**Background:**

Currently, there are a few treatment options for unresectable hepatocellular carcinoma (HCC) after progression following sorafenib (SOR) therapy, but with limited benefit. The purpose of this study was to investigate the efficacy and safety of tyrosine kinase inhibitors (TKIs) combined with immune checkpoint inhibitors (ICIs) as second-line treatment.

**Methods:**

From May 2018 to May 2021, a total of 93 HCCs who failed SOR treatment were included in this study and divided into TKI group (*n *= 37) and TKI-ICI group (*n *= 56). Overall survival (OS), progression-free survival (PFS), objective response rate (ORR), disease control rate (DCR) and safety were estimated among the two groups. In addition, univariate and multivariate Cox regression analyses were performed for OS and PFS to identify possible prognostic factors.

**Results:**

With a median follow-up time of 13.7 months, the median age of patients was 56 (range, 50–64) years and most were male. All of the patients were hepatitis virus-related HCC. Both median OS (7.63 months vs 19.23 months, *P *< 0.001) and median PFS (2.97 months vs 8.63 months, *P *< 0.001) were significantly improved in the TKI-ICI group compared to the TKI group. A significant increase in DCR was demonstrated in the TKI-ICI group compared to the TKI group (83.9% vs 45.9%, *P *= 0.0003), although no significant difference in ORR was reported (21.4% vs 8.1%, *P* = 0.1552). Multivariate Cox regression analysis of OS and PFS revealed that second-line regimen was an independent protective factor affecting death and progression in HCCs after SOR failure. In addition, Child–Pugh B7 was an independent risk factor of OS. Finally, there was no significant difference in the incidence of any grade or grade 3/4 adverse events (AEs) between the two groups, and no treatment-related deaths were observed.

**Conclusion:**

This real-world study suggests that the combination of TKIs and ICIs benefits more than mono-TKIs and is well tolerated in HCCs with hepatitis virus infection after SOR failure.

**Supplementary Information:**

The online version contains supplementary material available at 10.1007/s00262-022-03324-z.

## Introduction

Hepatocellular carcinoma (HCC) accounts for 75–85% of all liver cancers and is one of the most common and deadly cancers worldwide [1, 2]. HCC with early stage may be potentially curative, and the treatment options include hepatectomy, ablation and liver transplantation. However, HCC has an insidious onset, with more than 70% of patients being diagnosed initially at an advanced stage and requiring systemic therapy [2, 3]. Sorafenib (SOR) was the first tyrosine kinase inhibitor (TKI) recommended for unresectable HCC and was the only standard first-line treatment before 2017 [4, 5]. But only 30% of patients respond to this drug, the median time to progression (mTTP) is less than 4 months, and the incidence of adverse events (AEs) is high. Thus, SOR resistance poses a great challenge for subsequent treatment [6].

Regorafenib (REG) is one of the few treatment options for HCC who fails to first-line SOR [7]. And several studies showed HCCs with Eastern Cooperative Oncology Group performance status (ECOG PS) = 0, absence of microvascular invasion (MVI), Child–Pugh A and hyperbilirubinemia have a more pronounced survival benefit when REG is selected as second-line therapy [8–11]. Lenvatinib (LEN) was approved as a first-line TKI for advanced HCC following SOR, with significant improvements in outcomes except for median overall survival (mOS), which was not inferior to SOR [12]. Nevertheless, LEN has sustained similar treatment responses and toxicities in real-world studies between the TKI‐naïve and TKI‐experienced groups [13, 14]. The inhibition of FGFR-related signaling pathways may be involved in defeating the resistance of HCC to SOR [15, 16]. The advent of immune checkpoint inhibitors (ICIs) has provided new insights for second-line therapy, and nivolumab and pembrolizumab have demonstrated survival benefit in patients who have failed SOR [17, 18]. In addition, two other PD-1 inhibitors, camrelizumab (CAM) and sintilimab (SIN), are also effective and safe in the treatment of HCC in China [19, 20]. At present, anti-angiogenic drugs combined with immunotherapy have made great progress in advanced HCC, and atezolizumab plus bevacizumab, apatinib plus CAM and SIN plus IBI305 (anti-vascular endothelial growth factor (VEGF) drug) have shown good prospects in first-line treatment [21–23]. However, there are few studies of combination therapy in the second-line setting. One retrospective study found that REG combined with SIN had a better response than REG alone in the second-line treatment of advanced HCC, and the first-line drugs administered for the patients included LEN in addition to SOR [24].

Given the limited evidence for second-line combination therapy, we retrospectively investigated the efficacy and safety of the combination of LEN or REG plus ICIs in HCC after failure of first-line SOR and will give support for combination regimen in the second-line setting.

## Methods

### Patient

HCC who had failed first-line treatment with SOR at Beijing Ditan Hospital, Capital Medical University, from May 2018 to May 2021 was retrospectively enrolled. The main inclusion criteria were as follows: (1) age > 18 years; (2) at least one measurable target lesion according to modified Response Evaluation Criteria in Solid Tumors (mRECIST) criteria [25]; (3) ECOG PS 0–1; (4) Child–Pugh A or B ≤ 7. The main exclusion criteria were the following: (1) previous systemic therapy other than SOR monotherapy; (2) severe heart, brain, liver and kidney dysfunction; (3) coagulation disorders and bleeding tendency. Patients included were HCC progression assessed by mRECIST criteria after SOR treatment or discontinuation due to serious AEs, etc. Patients were allowed to undergo surgical and interventional procedures prior to and during the study. Patients were divided into groups according to the type of second-line medication.

### Treatment and evaluation

LEN was administered orally at initial doses of 12 mg/day and 8 mg/day, respectively, according to patients weighing ≥ 60 kg or < 60 kg. REG is used orally at an initial dose of 80 mg/day in four-week cycles for three weeks followed by one week off. Patients were administered SIN or CAM at 200 mg intravenously on day 1 of a 21-day therapy cycle after the first dose of LEN or REG. These agents were managed according to local regulations, and dose reductions or treatment interruptions were made in the event of tumor progression or unacceptable toxicity.

Second-line treatment was started after failure of SOR and response was assessed every 2–3 months by dynamic computed tomography (CT) or magnetic resonance imaging (MRI) based on mRECIST criteria. They were divided into complete response (CR), partial response (PR), stable disease (SD) and progressive disease (PD) [25]. And treatment-related AEs were recorded by Common Terminology Criteria for Adverse Events version 5.0 (CTC-AE 5.0). Primary outcomes included OS and progression-free survival (PFS) calculated from the start of second-line therapy; secondary outcome measures included objective response rate (ORR) and disease control rate (DCR) as well as safety.

The Ethics Committee of Beijing Ditan Hospital, Capital Medical University, approved the study, and it complied with the Declaration of Helsinki and clinical practice guidelines. All study patients provided informed written consent prior to study enrollment.

### Statistical analysis

Statistical analysis was performed using R software (4.0.5). Categorical variables were presented as numbers (percentages) and continuous variables with skewed distributions were presented as medians [interquartile range]. Pearson ‘s *χ*2 test or Fischer exact test was used to compare baseline characteristics, treatment response and AEs between groups. Survival analysis and comparison of OS and PFS were performed by Kaplan–Meier method and log-rank test. Univariate and multivariate Cox proportional hazards models were used to analyze prognostic factors for OS and PFS, and variables with *P* < 0.1 in univariate analysis were included in multivariate analysis. *P* < 0.05 was considered statistically significant.

## Results

### Patient baseline characteristics

**Fig. 1:**
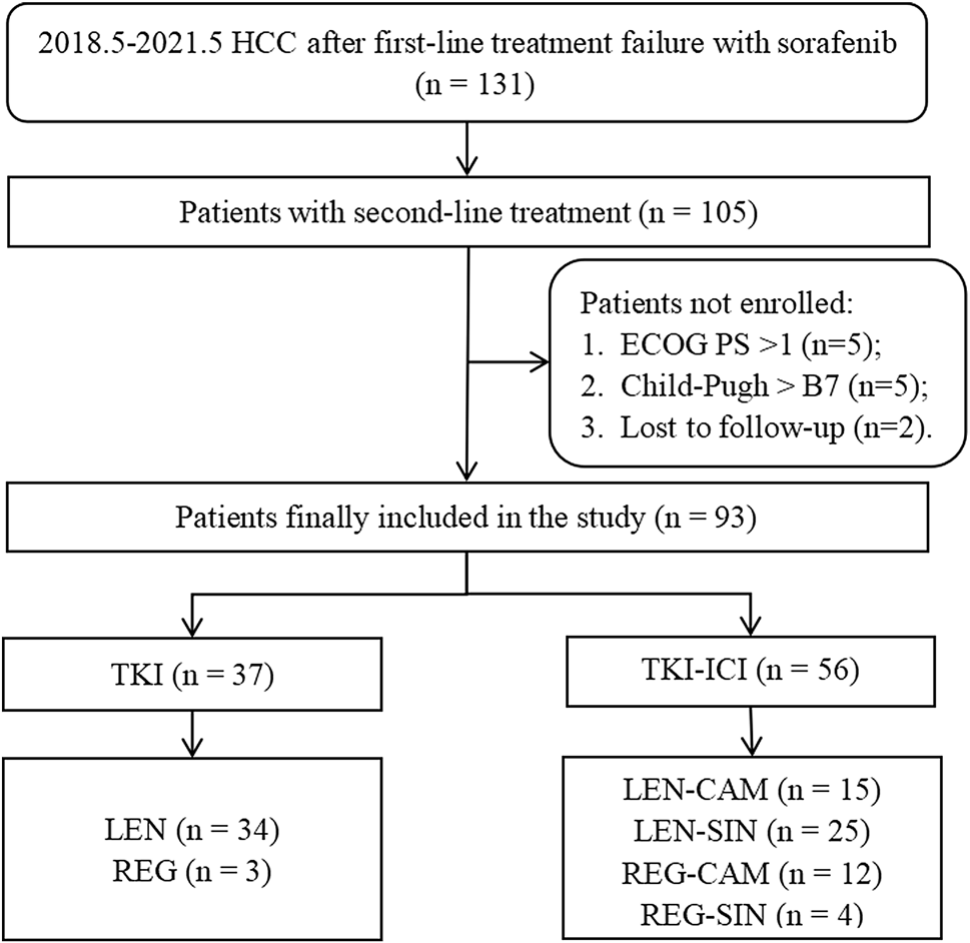
Flowchart of the study

From May 2018 to May 2021, a total of 131 HCCs discontinued SOR, which was administered as the first-line therapy, due to disease progression or serious AEs. Of whom, 105 patients received second-line systemic therapy, and 93 eligible patients were included in the study (Fig. 1). According to the type of drug used, patients were divided into two groups: 37 cases in the TKI monotherapy (TKI) group (3 REG and 34 LEN) and 56 cases in the TKI combined with immunotherapy (TKI-ICI) group (12 REG-CAM, 4 REG-SIN, 15 LEN-CAM and 25 LEN-SIN).

The median age was 56 (range: 50–64) years, 79 (84.9%) men. All of these HCC were caused by virus-related hepatitis, with hepatitis B virus (HBV) accounting for 91.4% (*n* = 85), hepatitis C virus (HCV) and HBV coinfection with HCV 4.3% (*n* = 4) each. Except for 4 patients with HBV-related HCC who did not receive antiviral therapy, the rest received nucleoside analogues, and the patients with HCV-related HCC received direct-acting antiviral drugs. Sixty-three (66.7%) patients had more than 3 intrahepatic tumors, 23 (24.7%) had tumors > 5 cm in maximum diameter, and 29 (31.2%) had Child–Pugh B7. In addition, less than half had portal vein tumor thrombus (PVTT) (*n* = 37, 39.8%) and extrahepatic metastases (*n *= 41, 44.1%). Overall, 77.4% of (*n* = 72) patients were in BCLC stage C. With regard to tumor treatment history, 21 (24.9%), 89 (95.7%) and 62 (66.7%) patients had previously undergone hepatectomy, transarterial chemoembolization (TACE) and ablation, respectively. There were no significant differences in clinical baseline data between the TKI and TKI-ICI groups, including disease stage and liver function (Table 1).

**Table 1:** Comparison of baseline characteristics

Characteristics	Overall (*n* = 93)	TKI (*n* = 37)	TKI-ICI (*n* = 56)	*P*
Age (years)	56 [50, 64]	53 [49, 60]	58.5 [51.5, 66.2]	0.0905
*Sex*				0.5816
Male	79 (84.9)	30 (81.1)	49 (87.5)	
Female	14 (15.1)	7 (18.9)	7 (12.5)	
*ECOG (%)*				0.1081
PS 0	33 (35.5)	9 (24.3)	24 (42.9)	
PS 1	60 (64.5)	28 (75.7)	32 (57.1)	
*Cause (%)*				0.7631
HBV	85 (91.4)	34 (91.9)	51 (91.1)	
HCV	4 (4.3)	2 (5.4)	2 (3.6)	
HBV + HCV	4 (4.3)	1 (2.7)	3 (5.4)	
Surgery (%)	21 (22.6)	6 (16.2)	15 (26.8)	0.3473
TACE (%)	89 (95.7)	35 (94.6)	54 (96.4)	1
Ablation (%)	62 (66.7)	29 (78.4)	33 (58.9)	0.0849
*Number (%)*				0.5153
≤ 3	30 (32.3)	10 (27.0)	20 (35.7)	
> 3	63 (67.7)	27 (73.0)	36 (64.3)	
*Size (%)*				0.8638
≤ 5 cm	70 (75.3)	27 (73.0)	43 (76.8)	
> 5 cm	23 (24.7)	10 (27.0)	13 (23.2)	
PVTT (%)	37 (39.8)	16 (43.2)	21 (37.5)	0.7358
Metastases (%)	41 (44.1)	17 (45.9)	24 (42.9)	0.936
*Child–Pugh (%)*				0.6598
Class A	64 (68.8)	24 (64.9)	40 (71.4)	
Class B7	29 (31.2)	13 (35.1)	16 (28.6)	
*BCLC (%)*				0.3473
Stage B	21 (22.6)	6 (16.2)	15 (26.8)	
Stage C	72 (77.4)	31 (83.8)	41 (73.2)	
*AFP (%)*				0.8693
≤ 400 ng/mL	55 (59.1)	21 (56.8)	34 (60.7)	
> 400 ng/mL	38 (40.9)	16 (43.2)	22 (39.3)	

*TKI* tyrosine kinase inhibitor, *ICI* immune checkpoint inhibitor, *ECOG PS* Eastern Cooperative Oncology Group performance status, *HBV* hepatitis B virus, *HCV* hepatitis C virus, *TACE* trans-arterial chemoembolization, *PVTT* portal vein tumor thrombosis, *BCLC* Barcelona Clinic Liver Cancer, *AFP* alpha-fetoprotein

### Survival analysis

**Fig. 2:**
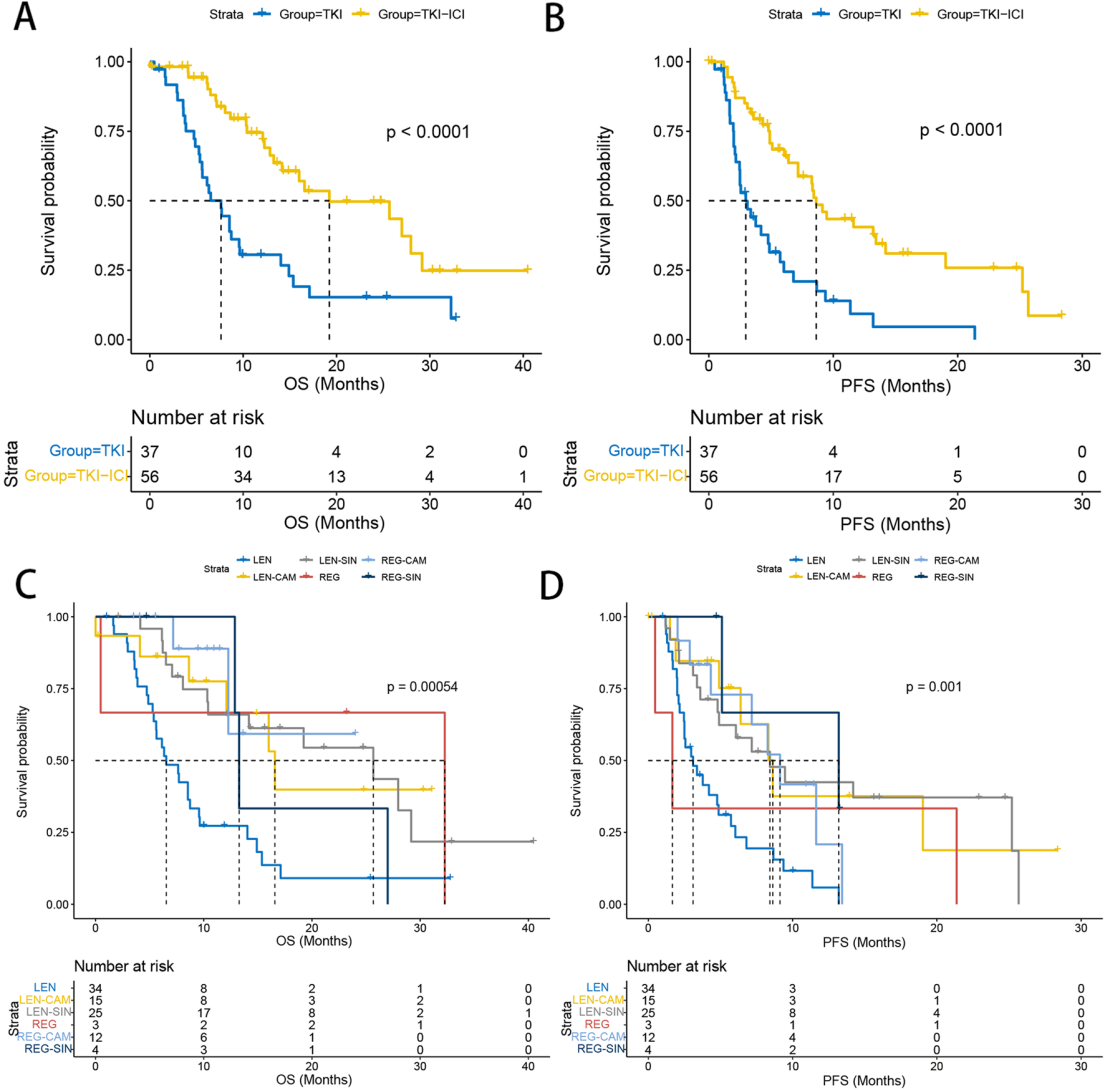
Kaplan–Meier curves of overall survival and progression-free survival between TKI group and TKI-ICI group and between different second-line treatment subgroups

At last follow-up, the median follow-up time was 13.7 months, 81.1% (*n* = 30) of patients had died, and 86.5% (*n* = 32) patients had progressed in the TKI arm. Meantime, 42.9% (*n* = 24) of patients had died and 58.9% (*n* = 33) patients had progressed in the TKI-ICI arm. Median OS (7.63 (95% CI, 5.6–14) months vs 19.23 (95% CI, 14.2—not reach (NR)) months, *P *< 0.001) was significantly prolonged in the TKI-ICI group compared to the TKI group. Again, this significant difference was observed in median PFS (2.97 (95% CI, 2.47–5.73) months vs 8.63 (95% CI, 7.17–19.03) months, *P *< 0.001) (Fig. 2A,B). We performed a more subdivided group considering the different therapeutic responses of different drugs. The TKI group contained REG and LEN, while the TKI-ICI group contained LEN + CAM, LEN + SIN, REG + CAM and REG + SIN. Figure 2C,D presents the survival curves and the median OS and PFS are documented in Table 2 of ESM. In addition, survival curves were plotted according to TKI and ICI drug, respectively (Supplementary Table 1). We found that median PFS and OS tended to be longer in REG than in LEN. The small number size of REG may limit the statistical power. In addition, no significant differences in survival were found between CAM and SIN.

### Therapeutic response

Changes in tumor target lesions according to mRECIST criteria were assessed for the study population, 1 (2.7%) CR, 2 (5.4%) PR, 14 (37.8%) SD and 20 (54.1%) PD in the TKI arm; 2 (3.6%) CR, 10 (17.9%) PR, 35 (62.5%) SD and 9 (16.1%) PD in the TKI-ICI arm. A significant increase in DCR was demonstrated in the TKI-ICI group compared to the TKI group (83.9% vs 45.9%, *P* = 0.0003), but the difference in ORR was not significant (21.4% vs 8.1%, *P* = 0.1552). Table 2 of ESM provides a detailed description of ORR and DCR across the groups.

### Cox regression analysis

**Fig. 3:**
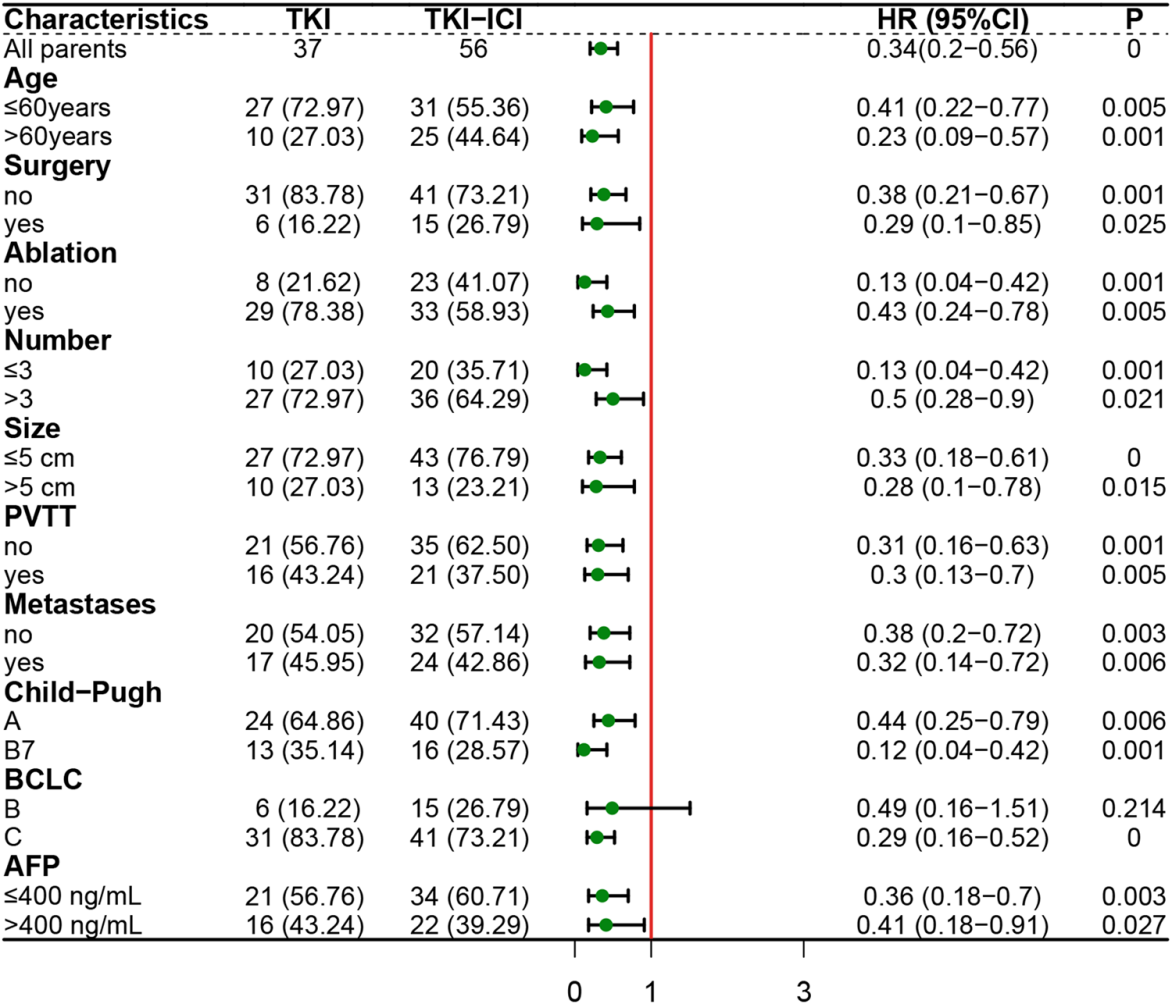
Subgroup analysis of progression-free survival in HCC after sorafenib failure between TKI group and TKI-ICI group

Univariate and multivariate Cox regression analyses using OS as the outcome showed that Child–Pugh B7 (HR, 2.31; 95% CI 1.2–4.44; *P* = 0.012) was an independent risk factor for death. Moreover, second-line TKI combined ICI (HR, 0.28; 95% CI 0.16–0.51; *P* < 0.001) was an independent favorable factor for OS compared with TKI (Table 2). In addition, regarding PFS, second-line treatment modality was the only independent predictor of HCC progression. TKI plus ICI (HR, 0.35; 95% CI 0.21–0.59; *P* < 0.001) was independently associated with longer PFS compared with TKI (Table 3). In addition, in the forest plot (Fig. 3), we observed an apparent survival advantage for the combination regimen versus mono-TKI in almost all subgroups including the subgroup of Child–Pugh B7, age > 60 years, AFP > 400 ng/mL. But the difference was not significant in HCC with BCLC stage B (HR, 0.49; 95% CI 0.16−1.51; *P* = 0.214).

**Table 2:** Cox proportional hazards model of prognosticators for OS

Characteristics	Univariate analysis		Multivariate analysis	
	*P*	HR(95%CI)	*P*	HR(95%CI)
Age (> 60 y vs ≤ 60 y)	0.579	0.85 (0.49–1.49)	–	–
Sex (male vs female)	0.673	0.84 (0.38–1.88)	–	–
ECOG (PS1 vs PS0)	0.327	1.35 (0.74–2.46)	–	–
Surgery (Yes vs No)	0.550	0.83 (0.44–1.55)	–	–
TACE (Yes vs No)	0.014	0.22 (0.06–0.73)	0.362	0.55 (0.15–2.01)
Ablation (Yes vs No)	0.676	1.14 (0.62–2.1)	–	–
*Second line*				
(TKI-ICI vs TKI)	< 0.001	0.34 (0.2–0.59)	< 0.001	0.28 (0.16–0.51)
Number (> 3 vs ≤ 3)	0.114	0.6 (0.32–1.13)	–	–
Size (> 5 cm vs ≤ 5 cm)	0.642	1.16 (0.62–2.18)	–	–
PVTT (Yes vs No)	0.569	1.17 (0.68–2.03)	–	–
Metastases (Yes vs No)	0.259	1.36 (0.8–2.33)	–	–
Child–Pugh (B7 vs A)	0.017	1.99 (1.13–3.49)	0.012	2.31 (1.2–4.44)
BCLC (C vs B)	0.131	1.74 (0.85–3.56)	–	–
AFP (> 400 ng/mL vs ≤ 400 ng/mL)	0.014	1.97 (1.15–3.38)	0.152	1.52 (0.86–2.68)

*OS* overall survival, *HR(95%CI)* hazard ratio(95% confidence interval), *ECOG PS* Eastern Cooperative Oncology Group performance status, *TACE* trans-arterial chemoembolization, *TKI* tyrosine kinase inhibitor, *ICI* immune checkpoint inhibitor, *PVTT* portal vein tumor thrombosis, *BCLC* Barcelona Clinic Liver Cancer, *AFP* alpha-fetoprotein

**Table 3:** Cox proportional hazards model of prognosticators for PFS

Characteristics	Univariate analysis		Multivariate analysis	
	*P*	HR(95%CI)	*P*	HR(95%CI)
Age (> 60 y vs ≤ 60 y)	0.279	0.75 (0.44–1.26)	–	–
Sex (male vs female)	0.321	0.99 (0.97–1.01)	–	–
ECOG (PS1 vs PS0)	0.061	1.69 (0.98–2.93)	0.168	1.48(0.85–2.58)
Surgery (Yes vs No)	0.293	0.73 (0.41–1.31)	–	–
TACE (Yes vs No)	0.509	1.95 (0.27–14.16)	–	–
Ablation (Yes vs No)	0.273	1.36 (0.78–2.36)	–	–
*Second line*				
(TKI-ICI vs TKI)	< 0.001	0.34 (0.2–0.56)	< 0.001	0.35(0.21–0.59)
Number (> 3 vs ≤ 3)	0.173	0.68 (0.4–1.18)	–	–
Size (> 5 cm vs ≤ 5 cm)	0.508	0.84 (0.49–1.42)	–	–
PVTT (Yes vs No)	0.891	0.97 (0.58–1.6)	–	–
Metastases (Yes vs No)	0.321	0.78 (0.47–1.28)	–	–
Child–Pugh (B7 vs A)	0.808	0.93 (0.53–1.64)	–	–
BCLC (C vs B)	0.476	0.81 (0.46–1.44)	–	–
AFP (> 400 ng/mL vs ≤ 400 ng/mL)	0.342	1.28 (0.77–2.13)	–	–

*PFS* progression-free survival, *HR(95%CI)* hazard ratio(95% confidence interval), *ECOG PS* Eastern Cooperative Oncology Group performance status, *TACE* trans-arterial chemoembolization, *TKI* tyrosine kinase inhibitor, *ICI* immune checkpoint inhibitor, *PVTT* portal vein tumor thrombosis, *BCLC* Barcelona Clinic Liver Cancer, *AFP* alpha-fetoprotein

### Safety analysis

All 93 patients were included in the safety analysis (Table 5 of ESM), and 79 (84.9%) patients experienced at least 1 AE. The most common AEs of any grade included hypertension, diarrhea, rash and nausea and vomiting. Most toxicities were mild to moderate, with approximately 1 in 5 patients experiencing grade 3/4 AEs and no treatment-related deaths. The overall incidence of AEs of any grade (78.4% vs 89.3%, *P* = 0.2528) and severe (18.9% vs 32.1%, *P* = 0.2424) was not significantly different between the two groups; there was also no significant difference in the incidence of different types of AEs. In addition, there were 5 cases of immune hepatitis, 1 case of immune myocarditis and 1 case of interstitial pneumonia in the TKI-ICI group regarding immune-related AEs. Most AEs were effectively ameliorated by dose modifications, and hypertension, hypothyroidism and immune-related AEs were greatly ameliorated by symptomatic treatment with antihypertensives, thyroxine and hormones. Intolerable discontinuation serious AEs occurred in very few patients (TKI group: hepatic encephalopathy in 1 patient; TKI-ICI group: immune hepatitis in 1 patient, severe hypertension in 1 patient).

## Discussion

In this study, we investigated possible second-line options in patients with HCC who were intolerant and progressed to SOR. The TKI arm included both LEN and REG, with a median PFS of 2.97 months, which was similar to the RESORCE trial [7], while shorter OS and lower DCR may be due to the inclusion of 31.2% of Child–Pugh B7 patients. In addition, the median OS in the TKI-ICI arm was 19.23 months, similar to IMbrave 150 trial [24], the difference was in the second-line setting. This study showed that TKI combined with ICI prolonged PFS by nearly 5 months, prolonged OS by more than 10 months. In addition, the combination regimen also had a greater improvement in tumor response compared with TKI alone. And TKI combined with ICI was an independent predictor of longer survivals in multivariate analysis for both OS and PFS. Our study suggested that TKI combined with ICI was more beneficial than mono-TKI not only in first-line treatment but also in second-line setting for advanced HCC with Child–Pugh A or B7.

As the first TKI, SOR significantly prolonged OS by almost 3 months, but only 30% of patients showed relative benefit, and resistance of HCC cells developed with continued treatment with SOR [4, 5]. Several global trials have been conducted following the introduction of SOR to overcome the compromised efficacy caused by SOR resistance, but unfortunately, these agents have not shown superiority. Until the emergence of REG, it is a standard second-line option after SOR treatment progression [7]. Similar survivals were observed in Lee et al. ‘s study [26]. And the benefit was more evident in another study of TACE plus REG, with a median PFS of 9.1 months [27]. LEN, although being approved as a first-line agent for HCC, has been found to be equally effective in different HCC treatment lines [13, 14]. Notably, the ORR in the TKI arm in this study was lower than the ORR in the LEN as second-line therapy reported above. The retrospective study by Hiraoka A et al. evaluated efficacy over 12 weeks in only 48.1% of patients and may have some selection bias [13]. In addition, there were more patients with PVTT and Child–Pugh B7 in the TKI group in our study compared to Chen et al. [14]. These factors, as important risk factors for response to cancer therapy, may contribute to the lower ORR. Compared to SOR, LEN and REG have more targets and have a potential role in overcoming SOR resistance. At the same time, the advent of immunotherapy provides new prospects for advanced HCC, and the combination of TKIs and ICIs has a synergistic effect [17–23]. REG enhances antitumor immunity by targeting the RET-Src axis to reduce PD-L1 and IDO1 expression and by promoting vascular normalization and increasing CD8 + T-cell infiltration and activation [28, 29]. In addition, LEN and nivolumab were not significantly different in HCC between the SOR‐naïve and SOR‐experienced groups, and prior nivolumab treatment failure was an independent factor for poor OS and PFS with the combination of LEN and pembrolizumab [30, 31]. Thus, target-immune combination therapy may become a potentially feasible second-line treatment for HCC.

Child–Pugh is the most commonly used classification systems to assess liver function status, and class A is associated with good liver function [32]. However, some patients following sorafenib failure tend to have worsening liver function [33]. In our study, Child–Pugh B7 patients accounted for 31.2%, and patients with B7 were strongly associated with worse OS compared with those with class A. Of note, there are limited data on the combination regimen of TKIs plus ICIs in patients with Child–Pugh B, but our study found that both Child–Pugh A and B7 could benefit from the combination regimen. In addition, no significant survival difference between the two groups was observed in the subgroup of BCLC stage B. It may be due to the small sample size (only 6 patients in TKI group and 15 patients in TKI-ICI group). The subsequent treatment options may also affect the survival since most of those patients have the opportunity to receive other systemic drugs or locoregional therapies.

Our retrospective study involved second-line agents including LEN, REG, CAM and SIN [7, 12, 19, 20], and we observed AEs consistent with those previously and no new toxicity profiles were identified. Hypertension, diarrhea and rash were the most common AEs of any grade or grade 3/4 in TKI group. The combination of TKIs plus ICIs did not significantly increase the incidence of AEs, including immune-related hepatitis, pneumonia and myocarditis.

Our study has some limitations, firstly, the retrospective design makes patient selection somewhat biased; secondly, the sample size was small and balanced matching between groups could not be performed; thirdly, different TKIs or PD-1 inhibitors have been administered for the patients; finally, the long enrollment time and the short follow-up time had not observed enough endpoint time. It is hoped that future studies will explore more appropriate second-line treatment options, while selecting convenient biomarkers and therapeutic subgroups to individualize treatment for patients.

## Conclusion

The combination of TKIs and ICIs for advanced HCC with hepatitis virus infection after SOR failure demonstrated survival benefits and was well tolerated, not only in patients with Child–Pugh A but also in those with Child–Pugh B7. Further prospective studies are warranted to validate this combination regimen.

### Supplementary Information

Below is the link to the electronic supplementary material.

Supplementary file1 (PDF 1901 KB)

Supplementary file1 (DOCX 17 KB)

## Data Availability

The dataset used for this study is available from the corresponding author upon reasonable request.

## Abbreviations

AE: Adverse event
CAM: Camrelizumab
CR: Complete response
DCR: Disease control rate
ECOG PS: Eastern Cooperative Oncology Group performance status
HBV: Hepatitis B virus
HCC: Hepatocellular carcinoma
HCV: Hepatitis C virus
ICI: Immune checkpoint inhibitor
LEN: Lenvatinib
mRECIST: Modified Response Evaluation Criteria in Solid Tumor
MVI: Microvascular invasion
ORR: Objective response rate
OS: Overall survival
PD: Progressive disease
PFS: Progression-free survival
PR: Partial response
PVTT: Portal vein tumor thrombus
REG: Regorafenib
SD: Stable disease
SIN: Sintilimab
SOR: Sorafenib
TACE: Transarterial chemoembolization
TKI: Tyrosine kinase inhibitor
TTP: Time to progression
VEGF: Vascular endothelial growth factor
