# Supplementary figures and images for: Treatment options for unresectable hepatocellular carcinoma with hepatitis virus infection following sorafenib failure
Source: Cancer Immunol Immunother. 2022 Nov 28;72(6):1395–403. doi: 10.1007/s00262-022-03324-z (PMC10198937; doi:10.1007/s00262-022-03324-z)

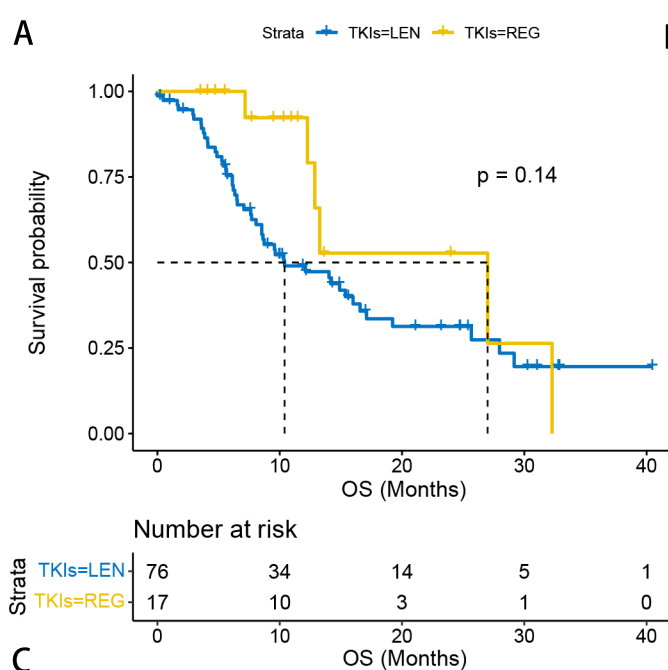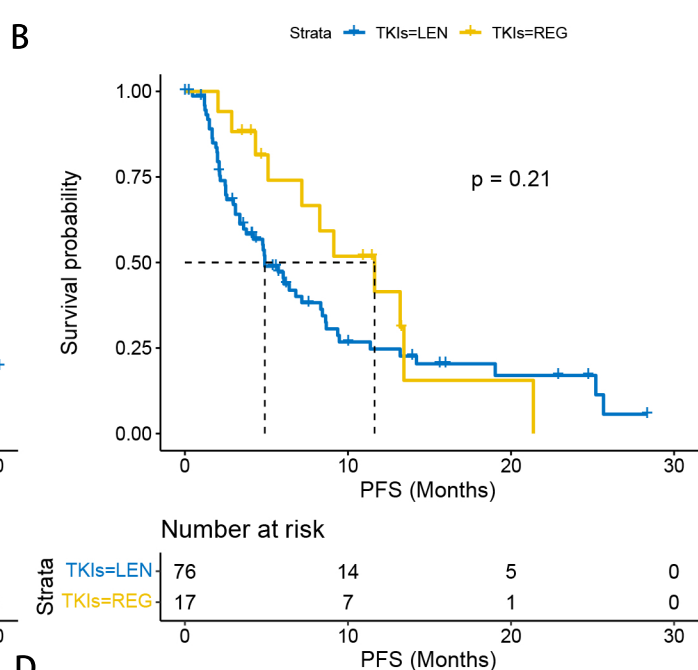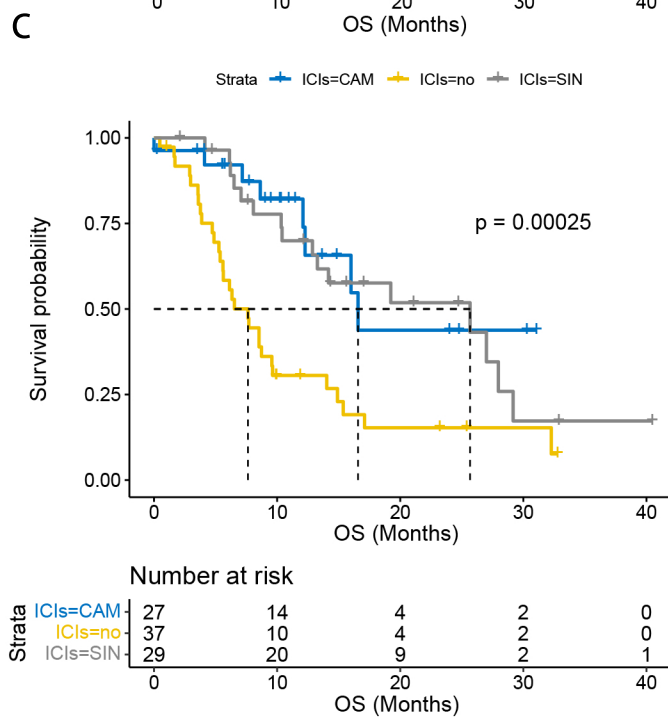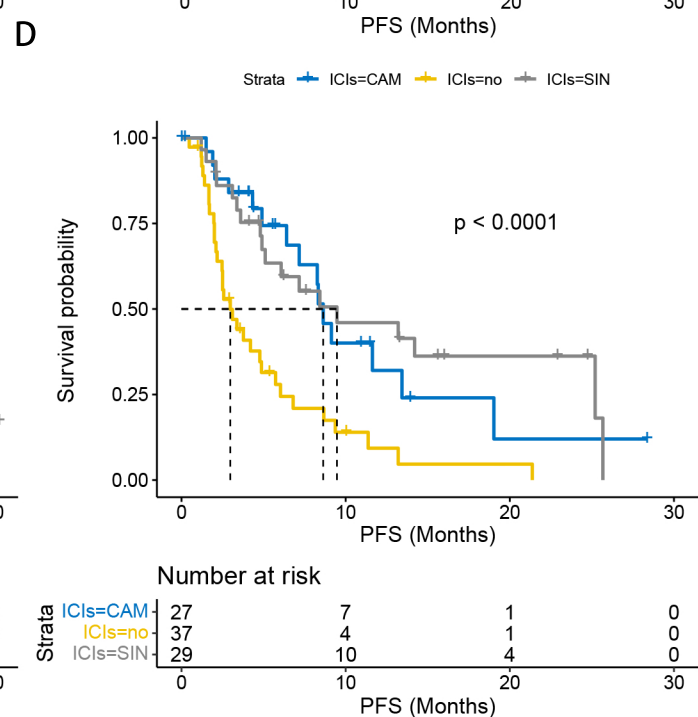

Supplement: Supplementary file 1 — Supplementary file1 (PDF 1901 KB) [file 262_2022_3324_MOESM1_ESM.pdf]
