# Supplementary material for: Treatment options for unresectable hepatocellular carcinoma with hepatitis virus infection following sorafenib failure
Source: Cancer Immunol Immunother. 2022 Nov 28;72(6):1395–403. doi: 10.1007/s00262-022-03324-z (PMC10198937; doi:10.1007/s00262-022-03324-z)
Supplement: Supplementary file 2 — Supplementary file1 (DOCX 17 KB) [file 262_2022_3324_MOESM2_ESM.docx]

| Second Line | ORR (%) | DCR (%) | mOS (months) | mPFS (months) |
| --- | --- | --- | --- | --- |
| LEN (n=34) | 2 (5.9) | 16 (47.1) | 6.53 (5.37-9.63) | 3.1 (2.47-5.73) |
| REG (n=3) | 1 (33.3) | 1 (33.3) | 32.27 (0.47-NR) | 1.67 (0.47-NR) |
| LEN-CAM (n=15) | 3 (20) | 13 (86.7) | 16.57 (12.10-NR) | 8.63 (6.40-NR) |
| LEN-SIN (n=25) | 7 (28) | 20 (80) | 25.67 (10.40-NR) | 8.43 (4.90-NR) |
| REG-CAM (n=12) | 1 (8.3) | 10 (83.3) | NR (12.27-NR) | 9.13 (7.17-NR) |
| REG-SIN (n=4) | 1 (25) | 4 (100) | 13.27 (12.87-NR) | 13.20 (5.10-NR) |
| TKI (n=37) | 3 (8.1) | 17 (45.9) | 7.63 (5.6-14) | 2.97 (2.47-5.73) |
| TKI-ICI (n=56) | 12 (21.4) | 47 (83.9) | 19.23 (14.2-NR) | 8.63 (7.17-19.03) |

**Table 2 Tumor response and survival between different agents**

ORR, objective response rate; DCR, disease control rate; mOS, median overall survival; mPFS, median progression-free survival; LEN, Lenvatinib; REG, Regorafenib; CAM, camrelizumab; SIN, sintilimab; TKI: tyrosine kinase inhibitor; ICI, immune checkpoint inhibitor; NR, not reach.

| Adverse Event | TKI group (n=37) | | TKI-ICI group (n=56) | | P |
| --- | --- | --- | --- | --- | --- |
|  | Any grade | Grade 3/4 | Any grade | Grade 3/4 |  |
| **Total AEs** | **29 (78.4)** | **7 (18.9)** | **50 (89.3)** | **18 (32.1)** | **0.2528** |
| Hypertension | 21 (56.8) | 4 (10.8) | 34 (60.7) | 9 (16.1) | 0.8693 |
| Nausea/vomiting | 12 (32.4) | 2 (5.4) | 19 (33.9) | 4 (7.1) | 1 |
| Fatigue | 11 (29.7) | 0 (0.0) | 17 (30.4) | 3 (5.4) | 1 |
| Diarrhea | 9 (24.3) | 3 (8.1) | 18 (32.1) | 5 (8.9) | 0.5621 |
| Rash | 5 (13.5) | 1 (2.7) | 13 (23.2) | 3 (5.4) | 0.373 |
| Decreased appetite | 5 (13.5) | 0 (0.0) | 9 (16.1) | 1 (1.8) | 0.967 |
| Proteinuria | 3 (8.1) | 0 (0.0) | 4 (7.1) | 1 (1.8) | 1 |
| Leukopenia | 2 (5.4) | 0 (0.0) | 5 (8.9) | 0 (0.0) | 0.819 |
| Hypothyroidism | 2 (5.4) | 0 (0.0) | 3 (5.4) | 1 (1.8) | 1 |
| Ascites | 2 (5.4) | 0 (0.0) | 4 (7.1) | 0 (0.0) | 1 |
| Hyperbilirubinemia | 1 (2.7) | 0 (0.0) | 2 (3.6) | 1 (1.8) | 1 |
| Thin | 1 (2.7) | 0 (0.0) | 2 (3.6) | 0 (0.0) | 1 |
| Thrombocytopenia | 1 (2.7) | 0 (0.0) | 2 (3.6) | 1 (1.8) | 1 |
| Hepatic encephalopathy | 1 (2.7) | 1 (2.7) | 1 (1.8) | 1 (1.8) | 1 |
| Gastrointestinal haemorrhage | 0 (0.0) | 0 (0.0) | 1 (1.8) | 1 (1.8) | 1 |
| **Immune-related AEs** | **0 (0.0)** | **0 (0.0)** | **7 (12.5)** | **5 (8.9)** | **0.0665** |
| Hepatitis | 0 (0.0) | 0 (0.0) | 5 (8.9) | 3 (5.4) | 0.1619 |
| Myocarditis | 0 (0.0) | 0 (0.0) | 1 (1.8) | 1 (1.8) | 1 |
| Interstitial pneumonia | 0 (0.0) | 0 (0.0) | 1 (1.8) | 1 (1.8) | 1 |

**Table 5 Treatment related adverse events**

TKI: tyrosine kinase inhibitor; ICI, immune checkpoint inhibitor.
